# Strain-level genomic variation of *Streptococcus mutans* and early childhood caries in preschool children from Northern Arizona and Hawaii

**DOI:** 10.7717/peerj.20808

**Published:** 2026-02-25

**Authors:** Ryann N. Whealy, Tara N. Furstenau, Alex Roberts, Jill Hager Cocking, Daryn Erickson, Breezy Brock, Rowan McCormick, Skylar Timm, Misty Pacheco, Summer Mochida-Meek, Viacheslav Fofanov

**Affiliations:** 1 Pathogen and Microbiome Institute, Northern Arizona University, Flagstaff, AZ, United States of America; 2 School of Informatics, Computing, and Cyber Systems, Northern Arizona University, Flagstaff, AZ, United States of America; 3 Department of Kinesiology and Exercise Sciences, University of Hawaii at Hilo, Hilo, HI, United States of America

**Keywords:** Early childhood caries, *Streptococcus mutans*, Health disparities, Microbial genetics, Genetic markers of virulence, Targeted amplicon sequencing

## Abstract

**Background:**

Early childhood caries (ECC) is the most common chronic disease of childhood, with especially high prevalence in Arizona and Hawaii where several racial and ethnic groups experience disproportionate burden. Although ECC is a polymicrobial disease, *Streptococcus mutans* plays a central role in its development, and evidence suggests that strain-level genetic variation influences its cariogenic potential. Understanding whether specific *S. mutans* lineages are associated with higher ECC risk and whether these lineages are more common in disproportionately affected groups is an important step toward identifying biological contributors to ECC outcomes.

**Methods:**

We conducted a cross-sectional study of 408 preschool-aged children (1–6 years) from Arizona and Hawaii. Saliva samples were tested for *S. mutans* using quantitative polymerase chain reaction (qPCR), and positive samples were genotyped using a custom amplicon sequencing assay. Logistic regression was used to evaluate associations between demographic factors (race, ethnicity, age, and sex), *S. mutans* colonization, and ECC status. To assess whether certain *S. mutans* genotypes were associated with ECC risk, we calculated a K-nearest-neighbor-smoothed risk score for each genotype based on patristic distances. Genetic markers of ECC risk were identified using a pseudo-genome-wide association approach.

**Results:**

ECC odds increased with age and were higher among Native Hawaiian/Pacific Islander, Asian, American Indian, and Hispanic children compared to non-Hispanic White children, although estimates for some groups were imprecise due to limited subgroups sizes. *S. mutans* colonization increased ECC odds by 361%, but colonization rates did not significantly differ across groups. Genotypes from Arizona and Hawaii showed no evidence of geographic clustering but ECC risk was non-randomly distributed across the phylogeny with multiple localized regions of higher risk genotypes. Native Hawaiian/Pacific Islander children were significantly more likely to carry higher-risk strains. Genetic markers linked with ECC risk mapped to genes involved in cariogenic processes—many of which were previously shown to be upregulated in caries-active plaque.

**Conclusions:**

The observed correlation between *S. mutans* genotype and ECC risk, together with the finding that higher-risk genotypes were more prevalent among at least one disproportionately affected group, suggests that strain-level variation may contribute to population-level disparities. The identification of functional markers linked to ECC risk further supports biologically meaningful strain-specific effects and warrants further investigation. These findings highlight the value of incorporating microbial genetic diversity into ECC risk frameworks, while recognizing that fully disentangling microbial contributions will require studies that integrate social, behavioral, and dietary determinants.

## Introduction

Early childhood caries (ECC) is the most common chronic childhood disease, affecting five times more children than asthma (Office of the Surgeon General (US), 2003). ECC is defined as the presence of one or more decayed, missing (due to caries), or filled tooth surfaces in any primary tooth in a child younger than six years (American Academy of Pediatric Dentistry, 2024). In the United States, approximately one in four children ages 2–5 experience ECC (Beltrán-Aguilar et al., 2005) which causes pain, difficulty eating, impaired nutrition, and missed school days (Krisdapong, Somkotra & Kueakulpipat, 2012; American Dental Association, 2021). Left untreated, ECC can progress to severe infections requiring urgent care, contributing to more than $1.6 billion in annual emergency dental costs (Dye, Arevalo & Vargas, 2010; Wall & Vujicic, 2015).

ECC arises from an ecological imbalance within the dental plaque biofilm. Frequent intake of sugary foods and drinks provides a constant supply of fermentable carbohydrates, which oral bacteria metabolize into organic acids. As acid accumulates and the biofilm pH drops, acid-producing and acid-tolerant organisms are selectively favored, shifting the community toward a cariogenic state that progressively demineralizes enamel and ultimately results in cavities (Marsh, 1994; Takahashi & Nyvad, 2011; Moimaz et al., 2016). Microbiome studies lend strong support to this ecological model, showing that oral microbial composition can accurately predict future cavity development well before the disease becomes clinically detectable (Teng et al., 2015; Grier et al., 2021). Across populations, multiple genera including *Streptococcus*, *Lactobacillus, Fusobacterium, Bifidobacterium, Scardovia, Actinomyces,* and *Veillonella* are consistently enriched in children who develop ECC (Li et al., 2007; Tanner et al., 2011).

Although no single microbe is both necessary and sufficient to cause caries, *Streptococcus mutans* remains one of the most well-established and consistent contributors (Loesche et al., 1975; Hamada & Slade, 1980; Loesche, 1986a; Loesche, 1986b; Lemos & Burne, 2008; Edlund et al., 2015; Bowen, 2016; Mazurel et al., 2025). Multiple cohort studies and a meta-analysis report that children colonized with *S. mutans* have roughly 3–4-fold higher odds of developing ECC, and a US based study reported effects as high as 8-fold even after adjusting for behavioral and demographic covariates (Ansai et al., 2000; Lynch et al., 2015; Edelstein, Ureles & Smaldone, 2016; Fan et al., 2019; Manchanda et al., 2023). *S. mutans* has high genomic diversity with an open pan-genome that influences cariogenic potential (Lembo et al., 2007; Zhang et al., 2009; Cornejo et al., 2013; Valdez et al., 2017; Meng et al., 2017; Bedoya-Correa, Rincón Rodríguez & Parada-Sanchez, 2019). *S. mutans* strains with higher virulence typically carry enhanced adhesion and biofilm genes (*e.g.*, glucan synthesis and SpaP adhesion variants) (Balakrishnan et al., 2002; Ren et al., 2016; Esberg et al., 2017; Matsumoto-Nakano, 2018; Zhang et al., 2021), collagen-binding adhesions (Cnm/Cbm) (Miller et al., 2015; Avilés-Reyes et al., 2017; Momeni et al., 2019), and elevated acid production and acid-tolerance systems (glycolysis/LDH and F_1_F_0_-ATPase) (Denepitiya & Kleinberg, 1984; Baker, Faustoferri & Quivey, 2017; Li et al., 2021; Zhang et al., 2021). However, many virulence genes are broadly distributed among children with or without disease (Argimón & Caufield, 2011; Argimón et al., 2014; Meng et al., 2017), indicating that allelic variants, regulatory elements, and accessory genes may modulate how these virulence factors are expressed and deployed in the oral environment (Esberg et al., 2017; Yang et al., 2019a).

Beyond the microbial processes that drive ECC, broader demographic, behavioral, environmental, and social factors influence a child’s risk for disease. Children from socially and economically disadvantaged backgrounds continue to experience high rates of ECC, even as overall caries prevalence has declined in the US over recent decades (Vargas & Ronzio, 2006; Luo et al., 2023). Nationwide, caries rates are nearly twice as high among Hispanic, Native American, and Native Hawaiian and Other Pacific Islander children compared to white children (Beltrán-Aguilar et al., 2005). Although disparities in access to dental care and other social determinants, such as income, education, language, and nativity, contribute to these patterns (Fisher-Owens et al., 2013; Han, 2019; Brega et al., 2020; Choi et al., 2023), they do not fully explain the elevated ECC rates observed in certain racial and ethnic groups (Park, Gomaa & Quinonez, 2022).

The persistence of ECC disparities has prompted growing interest in whether population-level differences in the oral microbiome contribute to differential susceptibility. Studies in adults demonstrate that oral microbial communities vary by race, ethnicity, geography, and socioeconomic background (Nasidze et al., 2009; Belstrøm et al., 2014; Gupta, Paul & Dutta, 2017; Yang et al., 2019b; Wang et al., 2024), and the few studies in children report race- and ethnicity-associated differences in alpha- and beta-diversity, community composition, and metatranscriptomic activity (Premaraj et al., 2020; Hsu et al., 2024). These findings raise the possibility that disparities in ECC may, in part, reflect differences in the microbial communities that children acquire or maintain. Given that *S. mutans* is both a key driver of ECC and a highly genetically diverse species, it is plausible that these same social, demographic, and geographic factors also influence which *S. mutans* lineages children are exposed to and the level of cariogenic risk they confer. Although strain-level comparisons across demographic groups remain limited, existing studies indicate that *S. mutans* lineages often cluster within families and, in some cases, within racial groups (Caufield et al., 1988; Caufield et al., 2007; Li & Caufield, 1995; Alaluusua et al., 1996; Li et al., 2005; Lynch et al., 2015; Momeni et al., 2018). Together, these observations suggest that circulating *S. mutans* strains may differ across populations and that variation in their cariogenic potential could contribute to the unequal ECC burden observed in certain groups.

Although several small studies have described family- or cohort-level clustering of *S. mutans* genotypes, to our knowledge, no large-scale work has evaluated strain-level differences across racial or ethnic groups or considered if certain genotypes are more common among these groups. To address this gap, this study focuses on children from Arizona and Hawaii, two high-prevalence regions with diverse populations. We asked four key questions: (1) Does ECC prevalence and *S. mutans* carriage differ across racial and ethnic groups within these regions? (2) Are *S. mutans* strains geographically clustered, or are they widely distributed across both locations? (3) Are specific *S. mutans* strains associated with higher ECC risk, and are these strains more common in disproportionately affected groups? (4) Which genetic markers in *S. mutans* that are linked to ECC risk? By integrating microbial genomics with demographic and geographic context, this study aims to identify strain-level *S. mutans* features that contribute to ECC risk and to determine whether some groups are more likely to harbor highly cariogenic strains. This work is an essential step toward clarifying the biological contributors to the disproportionate ECC burden observed in these communities.

## Materials & Methods

### Study population, sampling design, and screening procedures

Portions of this text were previously published as part of a preprint (Whealy et al., 2025). Children were recruited for sample collection from 14 participating preschool and daycare locations in Coconino County, Arizona and from five preschool locations in Hawaii. In Arizona, eligible children were between the ages of 1 and 5 years, and in Hawaii, children aged 1 to 6 years were included. No children were excluded based on race, ethnicity, sex, language, previous dental history, general health history, or recent antibiotic use. Four sampling campaigns were conducted in Arizona, lasting six months each, and were completed between Fall 2017 and Spring 2019 (Northern Arizona University Institutional Review Board 1105765-1). Two sampling campaigns were conducted in Hawaii and were completed between Fall 2021 and Spring 2022 (University of Hawaii at Hilo Institutional Review Board 2020-00156). This study used a rolling cross-sectional design, with continuous recruitment and sampling conducted across multiple campaigns. Although some children participated more than once, the study was not designed for longitudinal follow-up. Repeated samples from the same individual were not treated as independent observations in the statistical analyses.

School administrators opted into the study, and parents independently provided written informed consented to dental care services and sample collection. Parents were made aware that bacterial genomics were the focus of this study, and no human genetic material would be sequenced. Parents provided demographic information for their child, including age, gender, and ethnicity. The number and extent of caries-affected teeth (treated and untreated dental cavities) were assessed by trained dental hygienists through visual inspection under adequate lighting. Examiners recorded the presence of cavitated lesions, discoloration consistent with decay, and evidence of prior dental treatment. Screenings were conducted as part of the Coconino Public Health Services District’s Smart Smiles Oral Health Program in Northern Arizona or through the University of Hawaii at Hilo during school-based health screenings.

### Sample collection

While dental plaque is often used for microbial analysis in caries studies, we chose to use a less invasive saliva sampling method more appropriate for young children. *S. mutans* is frequently detected in saliva samples and has been shown to correlate with caries risk (Edelstein, Ureles & Smaldone, 2016). However, because saliva is a pooled, non-site-specific sample, it does not necessarily capture *S. mutans* strains associated with active cavity surfaces. Following the minimally invasive, standardized microbiome protocol developed by the Human Microbiome Project (HMP), a trained dental hygienist collected non-stimulated saliva by swabbing the buccal mucosa with a sterile ESwab (BD). Samples were stored on dry ice in one mL of modified liquid Amies medium during transport and then at −80 °C until processing.

### Quantitative polymerase chain reaction detection of *S. mutans*

Total DNA was extracted directly from 100 µL of transport media using the Quick-DNA Fungal/Bacterial Miniprep Kit (Zymo Research) following the manufacturer’s protocol. Samples were eluted in 100 µL of DNA elution buffer and stored at −80 °C until use. Nucleic acid concentration and purity were assessed using a NanoDrop spectrophotometer. The presence of *S. mutans* was confirmed using a previously established quantitative polymerase chain reaction (qPCR) assay (Yoshida et al., 2003) targeting the *gtfB* (GenBank accession M17361) gene using forward primer 5′-GCCTACAGCTCAGAGATGCTATTCT-3′, reverse primer 5′-GCCATACACCACTCATGAATTGA-3′, and probe 5′-FAM-TGGAAATGACGGTCGCCGTTATGAA-TAMRA-3′ with an expected amplicon size of 114 bp. Oligonucleotides were manufactured by Integrated DNA Technologies with standard desalting. Reactions were made in triplicate with a total volume of 10 µL and contained TaqMan Universal Master Mix (Thermo Fisher Scientific), one µL of template DNA, primers at a final concentration of 0.6 µM, and probes at 0.25 µM. The optimized cycle conditions for this reaction were: 50 °C for 2 min, 95 °C for 10 min, and 50 cycles of 95 °C for 15 s and 60 °C for 1 min using a QuantStudio 7 Flex Real-Time PCR System (Thermo Fisher Scientific). Each plate included quantified positive control genomic DNA (ATCC *S. mutans* 25175) and no-template controls (NTC). Cq values were determined using the Design and Analysis 2 software with automatic threshold and baseline settings. Samples with no amplification after 35 cycles were considered equivocal due to limited PCR success after this threshold, and samples with no amplification after 40 cycles were considered undetectable. Results only include runs where the positive control amplified as expected and no amplification was detected in the NTC.

### Development of amplicon sequencing assay

A targeted amplicon sequencing assay (AmpSeq) was developed to efficiently genotype *S. mutans* strains without requiring whole genome sequencing for all samples. This approach provided moderately high-resolution genotyping while remaining cost effective and scalable for this study. Amplicon targets were chosen using VaST v1.0.0 (Furstenau et al., 2018), which identifies a minimal set of target loci that maximize discrimination between genomes. The loci were chosen among 81,473 core genome single-nucleotide polymorphisms (SNPs) discovered from an alignment of 190 *S. mutans* genomes downloaded from NCBI complete genomes database (all assembly levels up to the year 2018) (O’Leary et al., 2016) using NASP v1.2.1 (Sahl et al., 2016) with NC_004350.2 as a reference (assembly accessions are provided in Table S1). From these, 29 amplicon targets covering 500 SNPs were selected to form the final panel, providing strong discriminatory power across the reference strains. A minimum spanning tree (GrapeTree; Zhou et al., 2018) of the reference genomes based on these targets is shown in Fig. S1. The average amplicon length (excluding primer regions) was 220 bp (range: 89–387 bp), and the total length of all amplicons was 6,834 bp. Primers were designed within conserved flanking regions to ensure robust amplification across diverse strains and were optimized for single-reaction multiplex polymerase chain reaction (PCR) (Table S2).

### Amplicon sequencing

DNA from *S. mutans* samples that tested positive with qPCR underwent further processing using the targeted amplicon sequencing assay. For the multiplex PCR, the KAPA 2G Fast Multiplex PCR Master Mix (Roche) was used with 29 *S. mutans-* specific primer pairs (Table S2). Primers were diluted to a final concentration of 0.2 µM. The optimized cycle profile was 95 °C for 3 min, 35 cycles of 95 °C for 15 s, 60 °C for 30 s, and 72 °C for 1 min 30 s, 72 °C for 1 min, and 10 °C indefinitely. Successful amplification was confirmed *via* gel electrophoresis, followed by a bead cleanup using a 2:1 ratio of AMPure (Beckman Coulter) beads. Barcodes were added through an extension PCR with the following cycle profile: 98 °C for 2 min, six cycles of (98 °C for 30 s, 60 °C for 20 s, and 72 °C for 30 s), 72 °C for 5 min, and 10 °C indefinitely. A second bead clean-up was performed with the same conditions. Libraries were quantified for pooling using a KAPA quantification kit, and the pooled libraries underwent fragment analysis for final quality assurance. Samples were sequenced on an Illumina MiSeq platform.

### Whole genome sequencing

Whole genome sequencing (WGS) was performed on a subset of *S. mutans-* positive samples (qPCR) from the northern Arizona cohort to validate the phylogenetic resolution of our AmpSeq assay. Sample swabs were streaked onto tryptone yeast extract cystine w/ sucrose & w/o bacitracin (TYCSB) media and incubation for 48 h at 37 °C. A single *S. mutans* colony, identified based on morphology, was isolated from each plate, and DNA was extracted using the Quick-DNA Fungal/Bacterial Miniprep Kit (Zymo Research). Sequencing was performed on an Illumina MiSeq instrument to produce 300 bp paired end reads. To compare the phylogenetic placement between samples that underwent parallel WGS and AmpSeq, we used WG-FAST (Sahl et al., 2015) to infer a maximum likelihood tree using SNPs called from this subset of samples and 190 reference genomes. Figure S2 shows that the AmpSeq and WGS-derived samples clustered consistently, indicating that our targeted AmpSeq panel effectively captured the phylogenetic signal observed in the WGS data.

### Sequence processing and analysis

Reads from both WGS and AmpSeq were quality trimmed and filtered using Fastp v0.20.1 (Chen, 2023) with default parameters. The sequences were aligned to reference *S. mutans* UA159 (NC_004350.2) using BWA-MEM v0.7.8 (Li, 2013) and SNPs were called using UnifiedGenotyper v3.4.46 (DePristo et al., 2011). SNPs within the expected amplicon regions (excluding primer regions) were concatenated and used to build a maximum likelihood tree with IQTree v1.6.12 (Nguyen et al., 2015) after filtering samples with less than 50% callable sites. IQTree was run with the best-fit model GTR+F+G4. There was a total of 173 sequences in the alignment and 250 SNPs (216 parsimony-informative and 34 singletons).

### Classifying *S. mutans* genotypes into caries risk groups using K-nearest neighbors

To test whether caries-associated *S. mutans* genotypes cluster along the phylogeny, we quantified each genotype’s local caries risk using a K-nearest neighbors (KNN) regression approach based on phylogenetic distance. Pairwise patristic distances were obtained from the maximum likelihood phylogeny using Dendropy v5.0.1 (Sukumaran & Holder, 2010). For each *S. mutans* genotype, we identified its five nearest phylogenetic neighbors (or more in the case of tied distances) and the KNN risk score was defined as the proportion of caries-positive samples among this neighborhood. For children with multiple *S. mutans* samples in the tree, their caries outcome was not counted twice within a neighborhood to avoid pseudo-replication. We assessed phylogenetic clustering by computing Pearson correlation between each genotype’s true binary ECC outcome (0: no cavities, 1: presence of at least one cavity, treated or untreated) and its KNN-derived risk score and compared this to a null distribution generated by 5,000 permutations of caries labels. The continuous KNN risk scores were then used to classify *S. mutans* genotypes into categorical risk groups: low (<25% caries-positive neighbors), medium (25–50%), and high (>50%). We defined three risk categories, rather than a binary classification, to allow a clearer distinction between genotypes associated with little to no disease and those with disease in a majority of neighboring cases.

### Statistical analysis

The original study targeted a sample size of approximately 400 preschool-aged children split between both locations and was sufficiently powered to determine whether *S. mutans* colonization was higher among children with ECC (>80% power, *a* = 0.05, two-sided). ECC status and *S. mutans* colonization were treated as binary variables (ECC: 0 = no cavities, 1 = presence of at least one cavity, treated or untreated; *S. mutans:* 0 = not detected, 1 = detected either by qPCR or amplicon sequencing in any sample). Race and ethnicity were used as covariates in the regression models and were categorized using indicator variables for Native American, Black, Native Hawaiian/Pacific Islander, Hispanic, Asian, and Non-Hispanic White with Non-Hispanic White serving as the reference group. KNN-based caries risk categories (low, medium, high) were coded numerically as 0, 1, and 2, respectively. For clade-based models, Clade 161 was selected as the reference group, as it was found exclusively in children within the low-risk category. Participant metadata and classifications are provided in Table S3.

To identify factors associated with the presence of at least one cavity, we performed logistic regression with the full model including age, race/ethnicity, sex, and *S. mutans* colonization; non-significant predictors were sequentially removed based on model fit. Penalized logistic regression was used to evaluate the association between clade membership and caries status, addressing issues of data separation due to unequal clade sizes. Logistic regression models were evaluated using receiver operating characteristic curves and AIC. To assess demographic associations with ECC risk categories, an ordinal logistic regression model was used with age, race/ethnicity, and sex as predictors. To test whether phylogenetic clustering of *S. mutans* strains was associated with geographic location (Hawaii *vs.* Arizona), we conducted a Mantel test using Scikit-Bio v0.6.2 (Pedregosa et al., 2011), applying Spearman correlation with 1,000 permutations between a genetic distance matrix (from the maximum likelihood tree) and a binary spatial distance matrix to represent each location.

### *S. mutans* genetic markers associated with caries risk

To investigate the genetic basis of caries risk among *S. mutans* genotypes, we conducted a pseudo-genome wide association study. Because our samples were sequenced at a limited number of loci, we could not assess genome-wide variation directly from our sample sequences. Instead, we used the phylogenetic placement of *S. mutans* reference genomes among our sequenced samples as a proxy to identify genetic features associated with high-risk clades. To do this, we drew a maximum likelihood tree that included our samples and 190 *S. mutans* reference genomes using IQ-Tree (Nguyen et al., 2015) from concatenated SNPs in the targeted amplicon regions. There were a total of 467 SNPs (340 parsimony-informative, 127 singletons) and the best-fit model was SYM+ASC+G4. The reference genomes were then classified into caries risk groups using the same KNN approach described above. From the classified reference genomes, we generated two types of input features for association testing: (1) a core genome SNP matrix (described above with 81,472 SNPs) and (2) a gene presence/absence matrix derived by annotating the genomes using Prokka v1.14.6 (Seemann, 2014) and performing core- and pan-genome analysis with Roary v3.13.0 (Page et al., 2015). Of the 9,806 genes identified, 788 were core genes (present in ≥ 99% of strains), 469 were soft core genes (in 95−99% of strains), 867 were shell genes (in 15–95% of strains), and 7,683 were cloud genes (in 0–15% of strains).

We applied two complementary approaches to identify associations between genetic features and ECC risk. We used extreme gradient boosting (XGBoost) (Chen & Guestrin, 2016), a gradient-boosted decision tree algorithm, to identify SNPs predictive of caries risk. SNP data (binary-encoded) and the predicted caries risk scores were analyzed using the XGBRegressor module from the XGBoost Python package v2.1.3 trained using 100 estimators. Feature importance was assessed using Shapley additive explanation (SHAP) values and were computed using the ‘shap’ Python library v0.46.0 (Lundberg & Lee, 2017). SNPs were ranked based on their mean absolute SHAP values, and the top 20 SNPs were annotated. Because this approach does not explicitly account for phylogenetic relationships among genomes, SNPs identified may reflect shared ancestry rather than independent, causal associations with the phenotype.

To control for population structure, our second approach used Pyseer v1.3.12 (Lees et al., 2018), which does account for shared ancestry between the genomes, so the features identified are more likely to be causally linked to caries risk. Using Pyseer, we analyzed the core genome SNPs and the presence/absence of 9,806 genes. The population structure was included by providing a distance matrix created using Dendropy v5.0.1 (Moreno, Holder & Sukumaran, 2024). Likelihood-ratio test *p*-values for gene and SNP associations were adjusted using a Bonferroni threshold defined as *α*/*M* where *α* = 0.05 and *M* is the total number of tests after quality control (3,461 for genes and 51,648 for SNPs). Prior to testing, features were filtered to retain only loci present in more than 1% of samples (Pyseer default), ensuring that invariant or extremely rare variants were excluded.

## Results

### Substantial ECC disparities across demographic groups in the study cohort

We collected saliva samples from 408 children, 266 from northern Arizona and 142 from Hawaii (Table 1). Caries data was available for 405 children and race/ethnicity was provided for 380 children. Among Non-Hispanic White children, only 9.5% (19/199) had at least one cavity (Table 2). In contrast, significantly higher rates of caries (Fisher’s Exact Test with Bonferroni correction for five comparisons) were observed among American Indian/Alaska Native (*p* = 0.004), Asian (*p* < 0.001), Hispanic (*p* = 0.005), and Native Hawaiian/Pacific Islander children (*p* < 0.001). Although Black and Native Hawaiian/Pacific Islander children had the highest prevalence of ECC (37.5% and 36.2%, respectively), the estimate for Black children was underpowered due to small sample size (*n* = 8) and therefore not statistically significant after correction (*p* = 0.04). Overall, these results show substantial disparities in ECC across these racial and ethnic groups.

**Table 1:** Demographics of participants. Total number of participants for each gender, age, and racial/ethnic group in the northern Arizona and Hawaii cohorts.

**Group**	**Category**	**Hawaii**	**Northern Arizona**	**Total**
Gender	Girl	66	128	194
	Boy	52	136	188
	Unknown	24	2	26
Age	1	0	30	30
	2	7	45	52
	3	37	73	110
	4	55	81	136
	5	19	33	52
	6	1	0	1
	Unknown	23	4	27
Race/Ethnicity	Non-Hispanic White	8	191	199
	Asian	50	4	54
	Black	2	6	8
	Hispanic	1	25	26
	Native American	0	35	35
	Native Hawaian/Pacific Islander	57	1	58
	Unknown	24	4	28

**Table 2:** ECC prevalence and *Streptococcus mutans* colonization across racial/ethnic groups in the study cohort. The table presents the total number of children sampled within each racial/ethnic group, the number and percentage of children with at least one cavity, and the number and percentage that tested positive for *S. mutans*.

Racial/Ethnic group	Total number of children	Children with caries	Percent caries positive	*S. mutans* colonized	Percent colonized
American Indian/Alaskan Native	35	10	28.6%	16	45.7%
Asian	54	16	29.6%	28	51.9%
Black	8	3	37.5%	3	37.5%
Hispanic	26	8	30.8%	12	46.2%
Native Hawaiian/Pacific Islander	58	21	36.2%	30	51.7%
Non-Hispanic White	199	19	9.6%	83	41.7%
Total	380	77	20%	172	45.3%

### *S. mutans* colonization is strongly associated with ECC but does not vary across demographic groups

*S. mutans* was detected in 44.9% (183) of children—114 (42.9%) from northern Arizona and 69 (48.6%) from Hawaii. Colonized children had a significantly higher rate of ECC compared with non-colonized children (33.5% *versus* 10.8%, respectively; chi-square test *p* < 0.0001). However, 29.9% (121/405) of children carried *S. mutans* but had no cavities at the time of sampling, and 5.9% (24/405) had cavities but no detectable *S. mutans.* Among children with reported race/ethnicity (Table 2), colonization rates did not differ across groups (chi-square test, *p* = 0.68). This indicates that while *S. mutans* colonization is strongly associated with disease at the individual level, we were unable to detect differences in colonization prevalence between demographic groups. Given the small sample sizes in several categories, the study had sufficient power to identify only large between group differences. Participant metadata, ECC status, and colonization status are provided in Table S3.

### Age, Race/Ethnicity, and *S. mutans* colonization predict ECC risk

Logistic regression was used to evaluate the risk factors associated with ECC while adjusting for all other variables. The odds of having at least one cavity increased by 80.09% for each additional year of age (*p* < 0.001; 95% CI [33.4–148.1]%) and by 360.49% for children with oral *S. mutans* colonization (*p* < 0.001; 95% CI [157.4–753.9]%). Significant differences in cavity risk were also observed across racial and ethnic groups, although some estimates were imprecise, as reflected by wide confidence intervals. Compared to White children, the odds were 714.20% higher for Black children (*p* = 0.020; 95% CI [26.6%–4,594.2%]), 330.94% higher for Native Hawaiian/Pacific Islander children (*p* < 0.001; 95% CI [100.6–837.2]%), 282.35% higher for Native American children (*p* = 0.006; 95% CI [45.3–880.7]%), 345.22% higher for Hispanic children (*p* = 0.005; 95% CI [52.6–1,151.5%]), and 214.68% higher for Asian children (*p* = 0.005; 95% CI [40.4–603.2]%). These findings indicate that age, race/ethnicity, and *S. mutans* colonization are each strong independent predictors of ECC risk, and the large disparities observed across racial and ethnic groups persist even after adjustment for other factors.

### *S. mutans* shows high genetic diversity and no geographic structure

**Figure 1:**
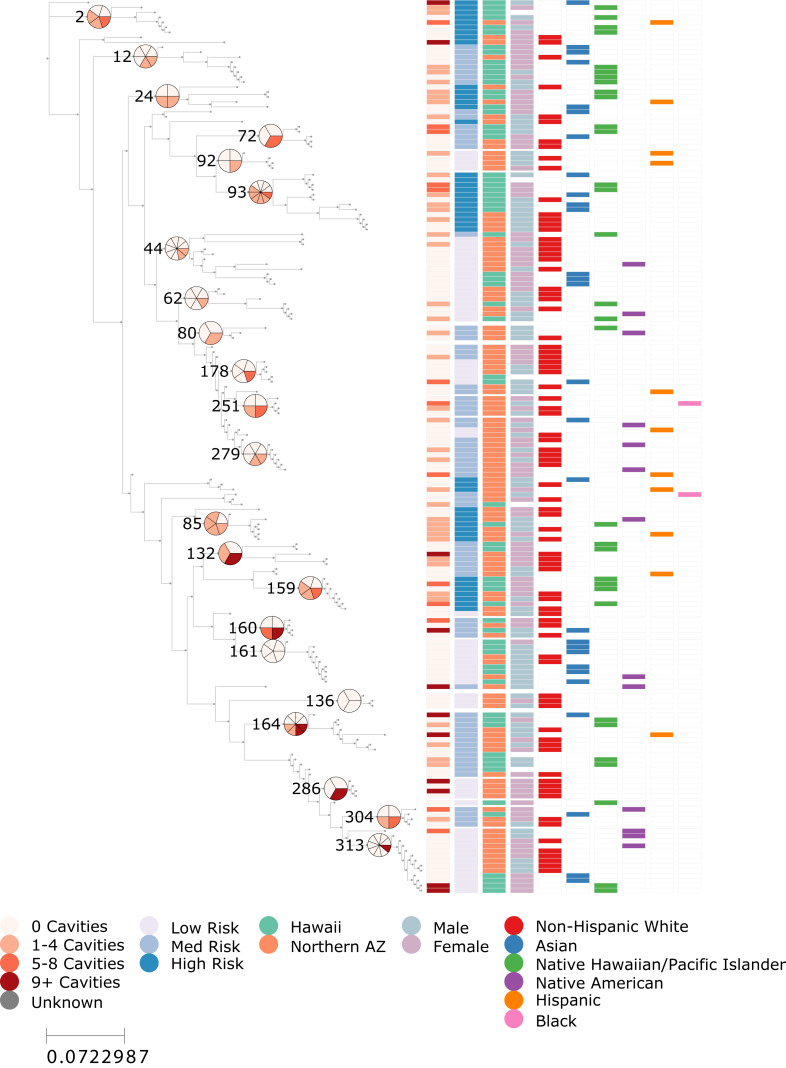
ECC risk is phylogenetically structured within *S. mutans*. Maximum likelihood phylogeny 176 *Streptococcus mutans* genotypes sequenced from children in Arizona and Hawaii. From left to right, the color bar columns indicate: (1) the number of cavities (treated or untreated) observed in the child from whom the sample was collected, (2) A K-nearest-neighbor-smoothed ECC risk score, calculated from the ECC status of the 5 closest genotypes in the tree (low <25%, medium = 25–50%, and high >50%), (3) sample location (Arizona or Hawaii), (4) sex of the child, and (5–10) binary indicators of self-reported race and ethnicity categories (Non-Hispanic White, Asian, Native Hawaiian/Pacific Islander, Native American, Hispanic, and Black). Select internal nodes of the tree are numbered to match clade identifiers used in clade-level logistic regression analyses. For these numbered clades, adjacent pie charts show the proportion of children with cavities among all children represented in that clade, using the same color scale as the first color bar column. Because a small number of children contributed more than one genotype, pie-chart proportions reflect participant-level deduplication to avoid overcounting individuals. The figure illustrates the phylogenetic structure of ECC risk but limited structuring by other demographic features including sample location.

We successfully generated 176 *S. mutans* sequences from 167 children—99 from northern Arizona and 68 from Hawaii. A small number of children contributed samples at more than one sampling event, meaning their genotypes may be slightly overrepresented in the phylogenetic dataset. Across the 6,834 bases targeted by PCR, we identified 250 SNPs, indicating substantial genetic diversity among sampled strains. Despite the broad geographic separation of the two study regions, we found no evidence of geographic clustering; genotypes from both locations were interspersed throughout the phylogeny and did not form region-specific clades (Fig. 1). A Mantel test showed only a very weak association between phylogenetic and geographical distance (*r* = 0.046, *p* = 0.011) indicating that regional barriers do not shape *S. mutans* genetic diversity in this population.

### *S. mutans* phylogeny reveals concentrated regions of elevated ECC risk

To test whether *S. mutans* genotypes were associated with ECC risk, we used a KNN-smoothed risk measure derived from patristic distances. The resulting low-, medium-, and high-risk classifications for each genotype are shown in Fig. 1 (second column of color bar). Visual inspection suggested that several regions of the phylogeny were enriched for higher risk genotypes, while others were dominated by low-risk strains. This pattern was statistically supported: continuous KNN risk scores were correlated with true caries outcomes (*r* = 0.379), showing a relatively strong association for a binary clinical phenotype in microbial population data. A permutation test confirmed that this level of local clustering was highly unlikely to arise by chance (*p* = 0.0002). These results demonstrate that ECC risk is not randomly distributed across the *S. mutans* phylogeny but instead exhibits clear local structure, with genetically similar strains tending to share similar disease outcomes.

We used Firth’s penalized logistic regression to test whether membership in any individual clade was independently associated with ECC. The tested clades are labelled on the tree in Fig. 1, with adjacent pie charts showing the proportion of caries-positive samples calculated with participant-level deduplication (*i.e.,* children with multiple samples contributed only one caries outcome). The model demonstrated good discriminative ability (AUC = 0.76), and several clades showed large estimated odds ratios relative to the low-risk reference clade, ranging from approximately 6- to 33-fold higher odds (Supplemental Data S1). Representative examples include Clade 2 (*p* = 0.055; OR = 15.4; 95% CI [0.95–2,405.9]), Clade 24 (*p* = 0.062; OR = 18.3; 95% CI [0.87–3,184.5]), Clade 85 (*p* = 0.013; OR = 33.0; 95% CI [1.90–5,507.8]), and Clade 93 (*p* = 0.030; OR = 15.9; 95% CI [1.26–2,292.3]). However, all estimates were accompanied by wide confidence intervals reflecting limited sample sizes, and none remained significant after correcting for multiple testing. Together with the strong local phylogenetic clustering detected in the KNN analysis, these findings suggest that multiple clades may contribute to elevated ECC risk but sparse sampling within individual clades prevented any one clade from reaching statistical significance.

### Native Hawaiian/pacific Islander children harbor higher-risk *S. mutans* genotypes

Because the KNN analysis revealed strong phylogenetic clustering of ECC risk, we next asked whether demographic factors might help explain why certain children carried higher-risk *S. mutans* genotypes. To do this, we used an ordinal logistic regression model to assess whether demographic factors predicted the KNN-assigned risk category (low, medium, or high) of their *S. mutans* genotypes. Native Hawaiian/Pacific Islander children were the only group that differed significantly from white children; their genotypes were assigned risk categories that were, on average, 0.46 points higher on a 0–2 scale (*p* = 0.004), corresponding to an expected risk category of 1.27. In contrast, sex and age were not significant predictors of KNN-based risk. Full model outputs are provided in Supplemental Data S1.

### *S. mutans* genetic features are associated with ECC risk

To identify genetic markers predictive of ECC risk, we performed a pseudo-genome wide association study using two complementary analytical approaches: XGBoost which detects lineage-linked predictors without accounting for population structure and Pyseer, which corrects for lineages effects to detect causal associations. With XGBoost, we identified the top 20 core genome SNPs with the highest feature importance, as determined by SHAP values (Figs. 3 and S4). The majority of these SNPs (15/20) were in coding regions (Table 3), while most of the non-coding SNPs were situated directly upstream or downstream of a coding region (4/5). Only one of the SNPs produced a non-synonymous mutation and this was in a multiple sugar transport system substrate-binding protein. Comparison with our previous metatranscriptomic study of plaque samples from children with ECC (Hsu et al., 2024), showed that 13 genes from these coding regions were significantly upregulated in cavity plaque, linking them to a functional role in caries formation.

**Table 3:** Top 20 *S. mutans* SNPs associated with ECC without lineage correction. The table shows annotations for SNPs identified using an XGBoost classification model trained to predict ECC status derived from our pseudo-genome-wide association study. SNPs are ranked from highest to lowest feature importance. Gene annotations are provided where available. The final column indicates whether the gene containing each SNP was previously found to be significantly upregulated in caries-active plaque in our previous metatranscriptomic study.

Position	Gene name	Ref. AA	Alt. AA	Mutation type	Description	Sig. up-regulated in caries in prior metatranscriptomic study
284143	polA	S	S	Syn	DNA polymerase I	Yes
1372446	estA	N	N	Syn	Alpha/beta hydrolase family protein, putative tributyrin esterase	Yes
1840238	SMU_RS08920	V	I	Ns	Multiple sugar transport system substrate-binding protein, extracellular solute-binding protein	Yes
1395913	SMU_RS10145	Q	K	Syn	ClbS/DfsB family four-helix bundle protein	
1130556	Non-Coding				85 bp upstream of GlsB/YeaQ/YmgE family stress response membrane protein	
1698048	SMU_RS08225	L	L	Syn	Nucleotidyltransferase	
1998269	yhgE	G	G	Syn	YhgE/Pip domain-containing protein, putative membrane protein	Yes
1338007	SMU_RS06415	A	A	Syn	Helix-turn-helix transcriptional regulator	
395860	rbfA	S	S	Syn	30S ribosome-binding factor RbfA	Yes
512055	murG	G	G	Syn	UDP-N-acetylglucosamine–N-acetylmuramyl-(pentapeptide) pyrophosphoryl-undecaprenol N-acetylglucosamine transferase	Yes
1226065	Non-Coding				20 bp downstream of zinc ABC transporter substrate-binding protein AdcA	Yes
446257	gmk	T	T	Syn	Guanylate kinase	Yes
332998	Non-Coding				22 bp upstream of purR pur operon repressor	Yes
342986	Non-Coding				77 bp upstream of gltB glutamate synthase large subunit	Yes
703137	Non-Coding					
1071837	ciaX	L	L	Syn	Three-component system regulator CiaX	
119888	SMU_RS00620	S	S	Syn	S-(hydroxymethyl)glutathione dehydrogenase/class III alcohol dehydrogenase	Yes
1840480	SMU_RS08925	E	E	Syn	Response regulator transcription factor	
319707	yidC1	N	N	Syn	Membrane protein insertase YidC1	Yes
1583154	livG	E	N	Syn	ABC transporter ATP-binding protein, branched-chain amino acid transport system ATP-binding protein	Yes

In the PySeer analysis, which included both SNPs and gene presence/absence data, multiple features were significantly associated with caries risk. We observed a strong negative association (= −0.273) for a gene annotated as *lagD*, which was present in eight of the genomes. This gene encodes a Lactococcin-G-processing and transport ATP-binding protein which plays a critical role in the production and export of lactococcin G, a bacteriocin with antimicrobial properties (Havarstein, Diep & Nes, 1995). The strong negative association suggests that the presence of this gene may reduce caries risk by modulating the microbial community. We also identified a strong negative association for a gene annotated as a helix-turn-helix-type transcriptional regulator CysL (= −0.2, present in 166 of the genomes) which is involved in sulfur metabolism with links to biofilm formation, oxidative stress resistance, and microbial interactions (Sperandio et al., 2010).

Pyseer also identified 165 significant core genome SNPs (Table S4), 46% (mean = 0.31) were positively associated with caries risk and 54% (mean = −0.27) were negatively associated. Most of the significant SNPs were in coding regions (87%) and 36% were non-synonymous. 60% of the SNPs were in genes that we previously found to be significantly upregulated in caries, and 41% of these were non-synonymous. SNPs in the same AraC-family helix-turn-helix transcriptional regulator gene (SMU_RS06415) were identified using both PySeer and XGBoost but they were at different positions. Other notable genes with significant non-synonymous SNPs included: *argG* (arginine metabolism and pH homeostasis), *nagA*, (carbohydrate metabolism), *gltB* (glutamate metabolism and pH regulation), *ftsY* (biofilm-related signal recognition receptor), *recN* (DNA repair and oxidative stress tolerance) and *pepX*, *pepF*, and *pepB* (peptide metabolism).

## Discussion

### Demographic differences in ECC prevalence and *S. mutans* oral colonization

Northern Arizona and Hawaii provided particularly relevant settings for this study because their ECC rates are among the highest in the US at approximately double the national average (Arizona Department of Health Services, 2009; Casamassimo et al., 2009; Dye et al., 2015). They also have high proportions of populations with disproportionately high risk of ECC: American Indian and Hispanic/Latino individuals in Arizona and Asian, and Native Hawaiian/Pacific Islander in Hawaii. Within this cohort, each of these groups showed significantly higher rates of ECC compared to white children and, although the magnitude of the effect had limited precision for some groups, the direction of association was broadly consistent with previous studies (Niendorff & Jones, 2000; Easa et al., 2005; Phipps et al., 2012; Warren et al., 2012; Fisher-Owens et al., 2013; Deguchi et al., 2013; Brega et al., 2020; Zheng et al., 2025). *S. mutans* colonization was significantly higher among children with ECC (detected in 72% of children with cavities), but colonization rates did not differ across racial/ethnic groups. Notably, ∼30% of *S. mutans* colonized children had not yet developed detectable caries and ∼6% of children had caries but no detectable *S. mutans.* This further reinforces that while *S. mutans* is a major contributor to ECC, it is not always necessary for caries development and is not always cariogenic when present (Socransky et al., 1998; Chase et al., 2004; Aas et al., 2008; Haffajee et al., 2008; Gross et al., 2010; Lemos et al., 2019; Lapirattanakul et al., 2020).

### *S. mutans* strain diversity, geographic distribution, and differential ECC risk

Our findings reinforce that *S. mutans* is a highly diverse species, yet this diversity showed little geographic structure. Genotypes from Arizona and Hawaii were broadly intermixed, and correlations between genetic and geographic distance were weak, consistent with prior work (Nakano et al., 2007). In contrast, ECC risk was non-randomly distributed across the phylogeny, with elevated risk concentrated in specific regions of the tree. Although several clades showed elevated odds ratios, sparse sampling across many lineages limited precision and statistical significance at the individual clade level. Nevertheless, these clade-level estimates aligned with the broader phylogenetic pattern, suggesting that cariogenic potential is structured by shared genetic background. Notably, Native Hawaiian/Pacific Islander children were disproportionately colonized by higher risk lineages and had elevated ECC burden. This enrichment raises the possibility that population-level disparities may partly reflect differential exposure or preferential retention of more virulent *S. mutans* strains. Such differences could arise through transmission networks, host-microbe compatibility, shared environmental conditions, or behavioral contexts unique to specific communities. Determining which of these processes drive the observed enrichment will be essential for understanding how microbial population structure intersects with ECC disparities.

### Identification of *S. mutans* genetic markers associated with ECC risk

We performed a pseudo-genome-wide association study to identify genetic features of *S. mutans* associated with elevated ECC risk. We used two complementary approaches that allowed us to capture lineage-dependent and lineage-independent associations. Significant genetic markers were associated with cariogenic processes including carbohydrate metabolism, signaling, pH regulation, stress tolerance, and biofilm formation. Variation in a gene encoding an AraC-family helix-turn-helix transcriptional regulator was independently identified in both of our analytic approaches (XGBoost and PySeer), making it a good candidate for further characterization. Transcription factors play a well-established role in *S. mutans* virulence (Abranches et al., 2018) and AraC-family regulators in *S. mutans* can modulate carbohydrate-utilization through systems such as the multiple sugar metabolism (*msm*) operon (Gallegos et al., 1997), which is directly relevant to cariogenicity. Importantly, 90% of the coding regions associated with significant markers in this study were also found to be significantly upregulated in caries plaque in our previous metatranscriptomic study (Hsu et al., 2024). This overlap suggests that the variants detected here are not only statistically associated with ECC risk but are also linked to functional pathways that are active in disease. Overall, we identified several high-priority genetic targets whose roles in virulence and strain-specific risk warrant further experimental validation.

### Study limitations

While this study provides valuable insights, there are some important limitations that affect the interpretation of our findings. Most notably, we did not collect socioeconomic, dietary, or oral hygiene data, nor did we assess individual susceptibility. Numerous studies show that frequent consumption of sugary snacks and beverages, inconsistent or inadequate toothbrushing, and poor parental supervision of oral hygiene are consistently associated with ECC (Marshall et al., 2003; Fisher-Owens et al., 2013; Thang Le et al., 2021), and these behaviors often intersect with broader structural barriers like limited access to dental care and lower health literacy, which are more prevalent in low-income and marginalized communities (Edelstein & Chinn, 2009; Warren et al., 2009). These unmeasured contextual factors may contribute to some of the group differences observed in our study. Future studies that integrate behavioral, socioeconomic, and host-level dimensions will be better positioned to fully characterize the multifactorial contributors to ECC risk.

## Conclusions

Pronounced ECC disparities were evident in our Northern Arizona and Hawaii populations, with American Indian/Alaska Native, Asian, Hispanic, and Native Hawaiian/Pacific Islander children exhibiting significantly higher disease prevalence than white children. *S. mutans* colonization was also strongly associated with ECC, but colonization rates were similar across groups and therefore could not account for these disparities. Rather, ECC risk varied across *S. mutans* lineages, with Native Hawaiian/Pacific Islander children more likely to harbor higher-risk strains—a pattern than may contribute to their elevated ECC rates. Importantly, these high-risk lineages were defined by biologically meaningful SNPs and gene variants, linking specific genomic features to strain-level cariogenic potential. Together, these findings highlight the importance of considering microbial genetic diversity, not just colonization status, when evaluating biological drivers of ECC disparities.

### Human ethics approval and consent to participate

This study was conducted in accordance with ethical guidelines for research involving human participants. Ethical approval for the Northern Arizona study was obtained from the Northern Arizona University Institutional Review Board (IRB #1105765-1), and approval for the Hawaii study was granted by the University of Hawaii at Hilo IRB (#2020-00156). Written informed consent was obtained from all participants or their legal guardians prior to sample collection. Clinical trial number: not applicable.

##  Supplemental Information

10.7717/peerj.20808/supp-1 Supplemental Information 1 Genetic diversity of *S. mutans* strains based on targeted amplicon sequencing The minimum spanning tree was constructed using SNPs called from sequences targeted in our amplicon sequencing assay. The tree illustrates the genetic distances between the samples sequenced as part of this study (blue) among 190 publicly available *S. mutans* reference genomes. The branch lengths are labelled and indicate the number of SNP differences between nodes. Each node is displayed as a pie chart sized by the number of genomes sharing an identical SNP profile. Where available, MLST types for the reference genomes are color coded (Do et al, 2009).

10.7717/peerj.20808/supp-2 Supplemental Information 2 The amplicon sequencing assay accurately recapitulates phylogenetic placement of *S. mutans* strains A maximum likelihood phylogeny was inferred using SNPs from both AmpSeq and WGS data for matched samples, alongside 190 publicly available *S. mutans* reference genomes. Each pair of samples (AmpSeq and WGS) is color coded. In every case, the AmpSeq and WGS results from the same sample cluster within the same clade. This consistent placement indicates that our AmpSeq assay captured a reliable phylogenetic signal.

10.7717/peerj.20808/supp-3 Supplemental Information 3 SHAP summary plot of the top 20 SNPs associated with ECC risk identified through XGBoost SHAP (Shapley additive explanations) values for the 20 SNPs with the highest feature importance in the XGBoost classifier. SNPs are ordered by overall contribution to model performance (top to bottom). Each point represents an individual sample, colored by SNP allelic state (blue=reference allele, red=alternative). Positive SHAP values indicate that the presence of the SNP increased the model-predictive ECC risk, whereas negative values indicate a decrease in ECC risk. SNPs showing greater spread or clearer separation between allelic states exert stronger influence on the model’s risk predictions.

10.7717/peerj.20808/supp-4 Supplemental Information 4 Heatmap of SHAP values for the top 20 SNPs identified by XGBoost SHAP values representing the modeled contribution of each of the top 20 SNPs to the predicted ECC risk for each sample. Rows correspond to SNPs (ranked by overall importance), and columns correspond to individual samples. Positive SHAP values (red) indicate that the SNP increased the model-predicted ECC risk, whereas negative values (blue) indicate a decrease in predicted risk. The magnitude of the value reflects the strength of the SNPs influence. The leftmost column shows the caries risk estimates that were used as the model’s target variable (yellow is high and dark blue is low), allowing comparison between predicted risk and the influence of individual SNPs.

10.7717/peerj.20808/supp-5 Supplemental Information 5 Reference sequence assembly accessions

10.7717/peerj.20808/supp-6 Supplemental Information 6 Primers used in AmpSeq assay The primers and their positions with the reference sequences are provided along with the expected amplicon sizes in the reference genome. The locus ID is provided for amplicons within or between coding regions.

10.7717/peerj.20808/supp-7 Supplemental Information 7 Participant metadata (1) unique, anonymized, participant IDs, (2) location of sample collection (Arizona or Hawaii), (3) age in years, (4) gender, (5) binary indicator for ECC (0=No caries, 1=one or more caries (treated or untreated)), (6) binary indicator for *S. mutans* detection (0=not detected, 1=detected in qPCR and/or amplicon sequencing in any sample), (7) ECC risk category based on KNN classification (0=low (¡25%), 1=medium (25–50%), and 2=high(¿50%)), (8) Race/Ethnicity classification, (9) clade ID for *S. mutans* sequences.

10.7717/peerj.20808/supp-8 Supplemental Information 8 *S. mutans* core genome SNPs significantly associated with ECC identified using PySeer with lineage correction

10.7717/peerj.20808/supp-9 Supplemental Information 9 Individual SRA accession numbers and metadata for sequencing data submitted as part of this project

10.7717/peerj.20808/supp-10 Supplemental Information 10 Minimum Information for Publication of Quantitative Real-Time PCR Experiments Checklist

10.7717/peerj.20808/supp-11 Supplemental Information 11 Raw statistical output tables

## Additional Information and Declarations

### Data Availability

The following information was supplied regarding data availability: The data is available in the Supplemental Files. The raw sequence data are available at NCBI BioProject: PRJNA1225904.
