# Supplementary figures and images for: Strain-level genomic variation of Streptococcus mutans and early childhood caries in preschool children from Northern Arizona and Hawaii
Source: PeerJ. 2026 Feb 25;14:e20808. doi: 10.7717/peerj.20808 (PMC12949586; doi:10.7717/peerj.20808)

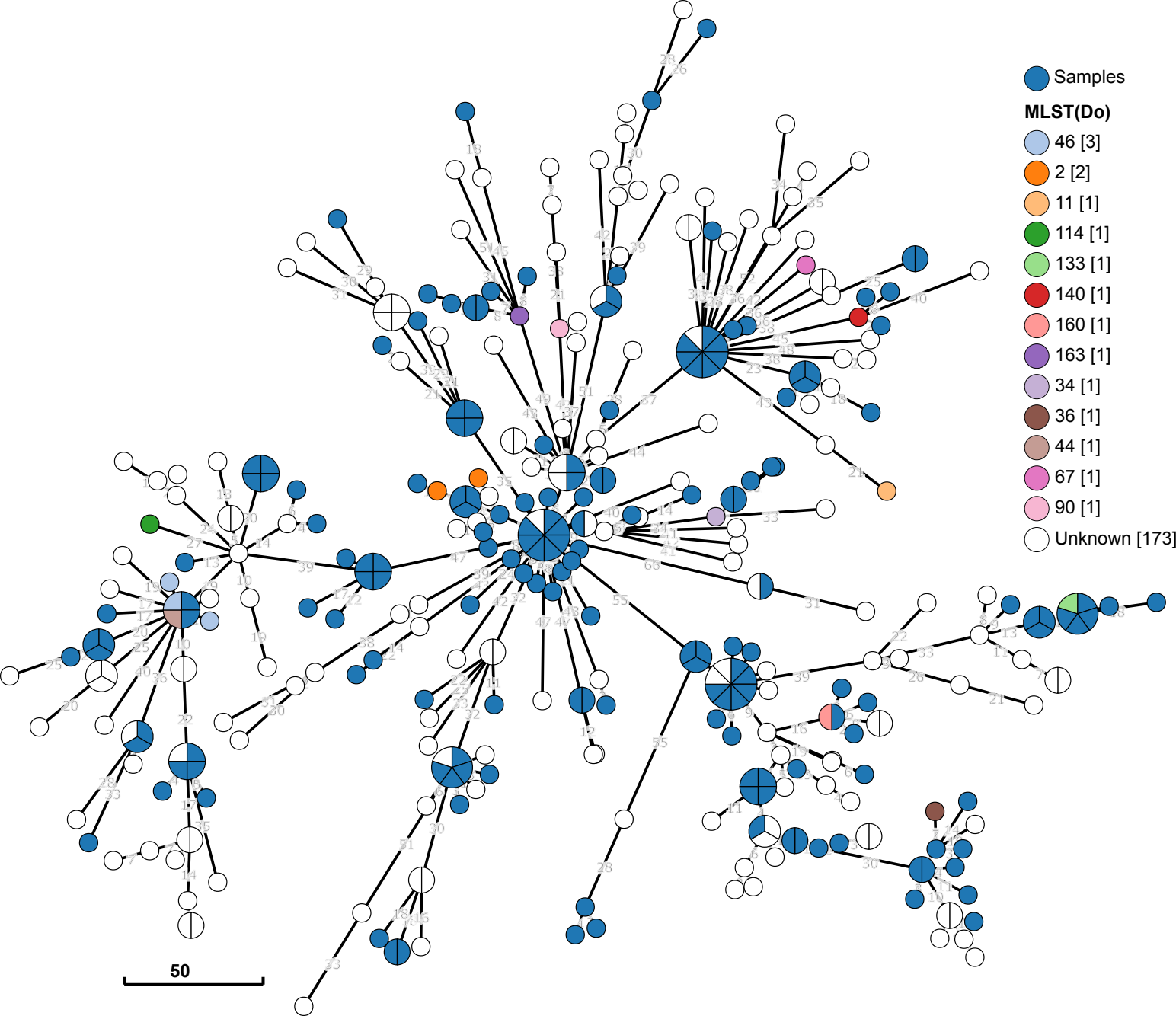

Supplement: Supplemental Information 1 — The minimum spanning tree was constructed using SNPs called from sequences targeted in our amplicon sequencing assay. The tree illustrates the genetic distances between the samples sequenced as part of this study (blue) among 190 publicly available S. mutans reference genomes. The branch lengths are labelled and indicate the number of SNP differences between nodes. Each node is displayed as a pie chart sized by the number of genomes sharing an identical SNP profile. Where available, MLST types for the reference genomes are color coded (Do et al, 2009). [file peerj-14-20808-s001.pdf]

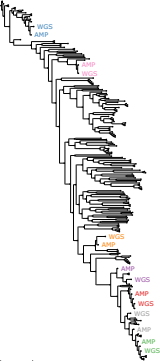

0.0755417

Supplement: Supplemental Information 2 — A maximum likelihood phylogeny was inferred using SNPs from both AmpSeq and WGS data for matched samples, alongside 190 publicly available S. mutans reference genomes. Each pair of samples (AmpSeq and WGS) is color coded. In every case, the AmpSeq and WGS results from the same sample cluster within the same clade. This consistent placement indicates that our AmpSeq assay captured a reliable phylogenetic signal. [file peerj-14-20808-s002.pdf]

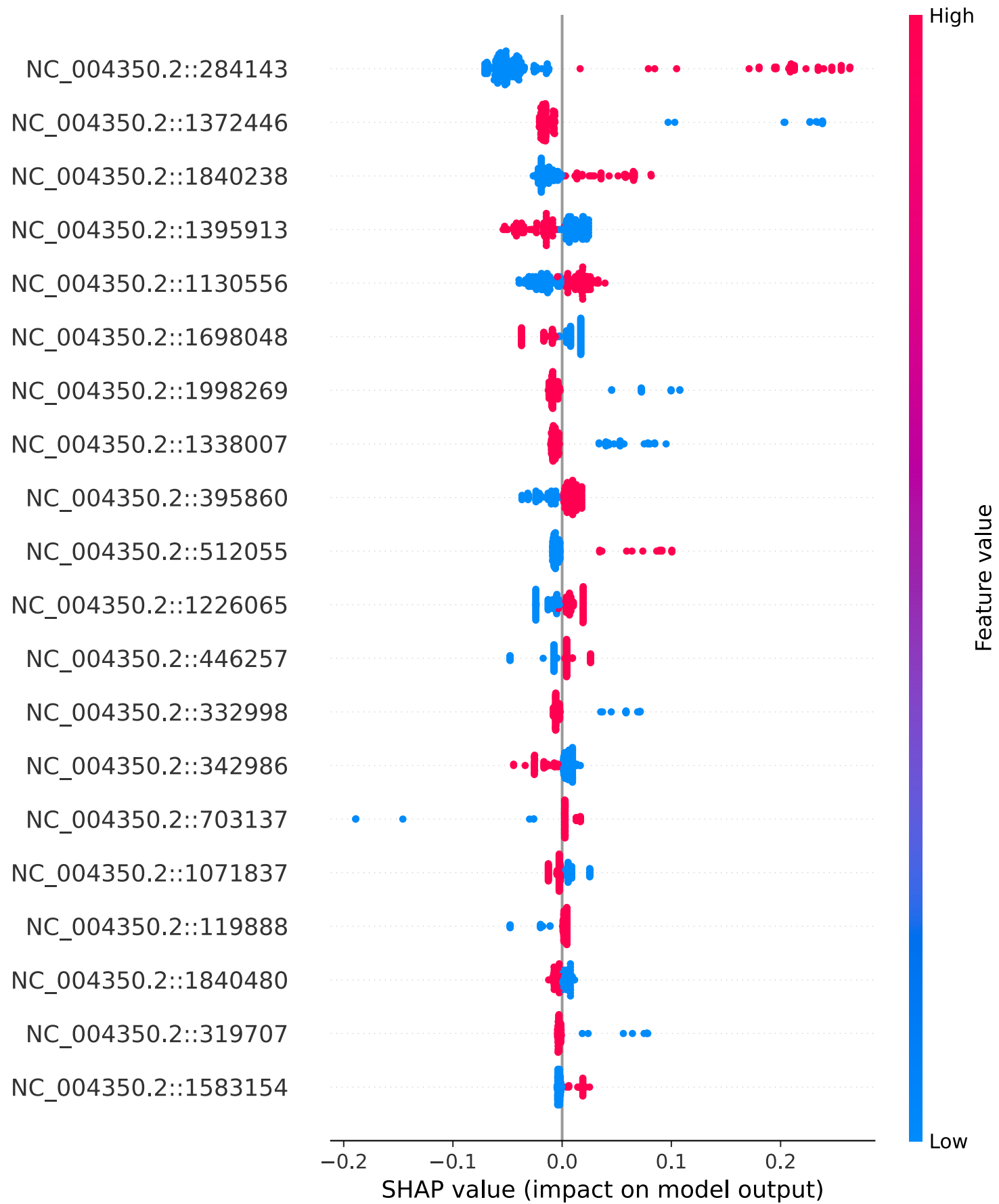

Supplement: Supplemental Information 3 — SHAP (Shapley additive explanations) values for the 20 SNPs with the highest feature importance in the XGBoost classifier. SNPs are ordered by overall contribution to model performance (top to bottom). Each point represents an individual sample, colored by SNP allelic state (blue=reference allele, red=alternative). Positive SHAP values indicate that the presence of the SNP increased the model-predictive ECC risk, whereas negative values indicate a decrease in ECC risk. SNPs showing greater spread or clearer separation between allelic states exert stronger influence on the model’s risk predictions. [file peerj-14-20808-s003.pdf]
