# Supplementary material for: Strain-level genomic variation of Streptococcus mutans and early childhood caries in preschool children from Northern Arizona and Hawaii
Source: PeerJ. 2026 Feb 25;14:e20808. doi: 10.7717/peerj.20808 (PMC12949586; doi:10.7717/peerj.20808)
Supplement: Supplemental Information 4 — SHAP values representing the modeled contribution of each of the top 20 SNPs to the predicted ECC risk for each sample. Rows correspond to SNPs (ranked by overall importance), and columns correspond to individual samples. Positive SHAP values (red) indicate that the SNP increased the model-predicted ECC risk, whereas negative values (blue) indicate a decrease in predicted risk. The magnitude of the value reflects the strength of the SNPs influence. The leftmost column shows the caries risk estimates that were used as the model’s target variable (yellow is high and dark blue is low), allowing comparison between predicted risk and the influence of individual SNPs. [file peerj-14-20808-s004.pdf]

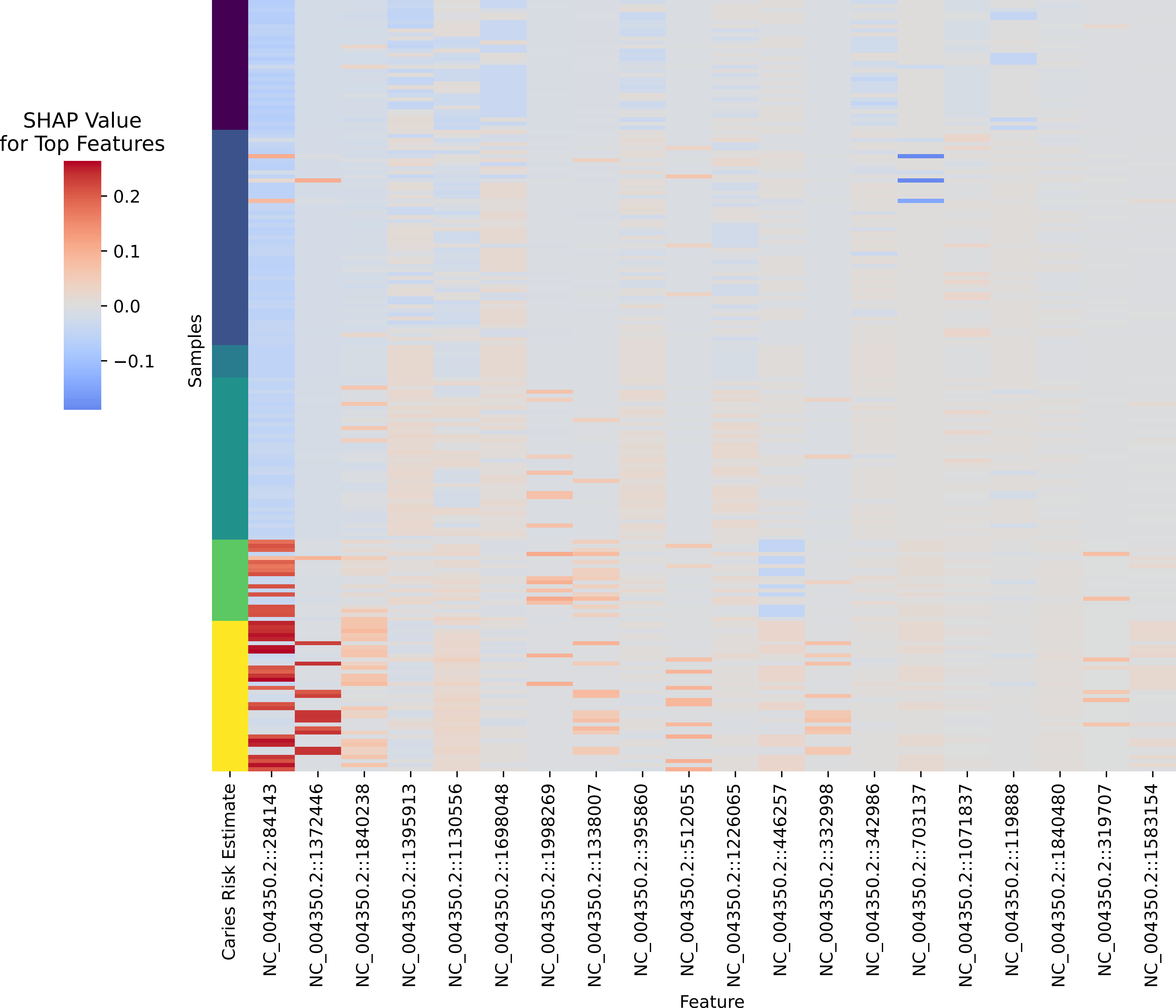
SHAP Value
for Top Features
0.2
0.1
0.0
−0.1
Samples
Caries Risk Estimate
NC_004350.2::284143
NC_004350.2::1372446
NC_004350.2::1840238
NC_004350.2::1395913
NC_004350.2::1130556
NC_004350.2::1698048
NC_004350.2::1998269
NC_004350.2::1338007
NC_004350.2::395860
NC_004350.2::512055
NC_004350.2::1226065
NC_004350.2::446257
NC_004350.2::332998
NC_004350.2::342986
NC_004350.2::703137
NC_004350.2::1071837
NC_004350.2::119888
NC_004350.2::1840480
NC_004350.2::319707
NC_004350.2::1583154
Feature
