# Supplementary material for: Strain-level genomic variation of Streptococcus mutans and early childhood caries in preschool children from Northern Arizona and Hawaii
Source: PeerJ. 2026 Feb 25;14:e20808. doi: 10.7717/peerj.20808 (PMC12949586; doi:10.7717/peerj.20808)
Supplement: Supplemental Information 11 [file peerj-14-20808-s011.docx]

##
## Call:
## glm(formula = Cavities ~ Age + race + MutansPosQPCR_AMPSEQ, family = binomial(link = "logit"),
## data = results.2)
##
## Coefficients:
## Estimate Std. Error z value Pr(>|z|)
## (Intercept) -5.1546 0.6853 -7.521 5.43e-14 ***
## Age 0.5883 0.1578 3.729 0.000192 ***
## raceAsian 1.1464 0.4088 2.804 0.005041 **
## raceBlack 2.0970 0.9029 2.322 0.020209 *
## raceHisp 1.4934 0.5316 2.809 0.004963 **
## raceNAAI 1.3412 0.4832 2.776 0.005509 **
## raceNHPI 1.4608 0.3915 3.731 0.000191 ***
## MutansPosQPCR_AMPSEQ1 1.5271 0.3046 5.013 5.37e-07 ***
## ---
## Signif. codes: 0 '***' 0.001 '**' 0.01 '*' 0.05 '.' 0.1 ' ' 1
##
## (Dispersion parameter for binomial family taken to be 1)
##
## Null deviance: 382.61 on 378 degrees of freedom
## Residual deviance: 305.00 on 371 degrees of freedom
## (29 observations deleted due to missingness)
## AIC: 321
##
## Number of Fisher Scoring iterations: 5

## Waiting for profiling to be done...

## 2.5 % 97.5 %
## (Intercept) -6.5848261 -3.8905471
## Age 0.2881996 0.9085594
## raceAsian 0.3394628 1.9504131
## raceBlack 0.2356948 3.8489105
## raceHisp 0.4227298 2.5269382
## raceNAAI 0.3739174 2.2830718
## raceNHPI 0.6960762 2.2377372
## MutansPosQPCR_AMPSEQ1 0.9456114 2.1446416

## OddsRatio OR_lower95 OR_upper95
## (Intercept) 0.005772741 0.001381167 0.02043416
## Age 1.800858276 1.334023557 2.48074619
## raceAsian 3.146772106 1.404193023 7.03159156
## raceBlack 8.141966374 1.265787929 46.94189272
## raceHisp 4.452201976 1.526121935 12.51512825
## raceNAAI 3.823548234 1.453417119 9.80675854
## raceNHPI 4.309378993 2.005866622 9.37209999
## MutansPosQPCR_AMPSEQ1 4.604876775 2.574386887 8.53898006

## PercentChange PC_lower95 PC_upper95
## (Intercept) -99.42273 -99.86188 -97.95658
## Age 80.08583 33.40236 148.07462
## raceAsian 214.67721 40.41930 603.15916
## raceBlack 714.19664 26.57879 4594.18927
## raceHisp 345.22020 52.61219 1151.51283
## raceNAAI 282.35482 45.34171 880.67585
## raceNHPI 330.93790 100.58666 837.21000
## MutansPosQPCR_AMPSEQ1 360.48768 157.43869 753.89801

## Registered S3 method overwritten by 'logistf':
## method from
## nobs.logistf MuMIn

## logistf::logistf(formula = Cavities ~ Clade2, data = risk.2)
##
## Model fitted by Penalized ML
## Coefficients:
## coef se(coef) lower 0.95 upper 0.95 Chisq p
## (Intercept) -2.3978953 1.477098 -7.27518334 -0.2215505 4.87573437 0.02723685
## Clade22 2.7343675 1.693379 -0.05189156 7.7856871 3.68398905 0.05493727
## Clade212 1.8101086 1.674527 -1.00799075 6.8377336 1.47868405 0.22398053
## Clade224 2.9087209 1.802355 -0.13447377 8.0660582 3.48274834 0.06201167
## Clade244 1.2992830 1.647772 -1.46315378 6.3035109 0.75374740 0.38529237
## Clade262 1.0986123 1.740777 -2.05167915 6.1718363 0.44624903 0.50412164
## Clade272 1.8870696 1.802355 -1.36705766 7.0201279 1.27351077 0.25910909
## Clade280 1.8870696 1.802355 -1.36705766 7.0201279 1.27351077 0.25910909
## Clade285 3.4965076 1.752343 0.64208897 8.6139244 6.11058485 0.01343747
## Clade292 1.5505974 1.770367 -1.65105598 6.6517090 0.88039007 0.34809486
## Clade293 2.7656201 1.599339 0.23307460 7.7373133 4.70920682 0.03000149
## Clade2132 2.9087209 1.802355 -0.13447377 8.0660582 3.48274834 0.06201167
## Clade2136 0.4519851 2.113654 -4.87880089 5.7867551 0.04546638 0.83114876
## Clade2159 2.3978953 1.726794 -0.51915821 7.4746607 2.51745400 0.11259224
## Clade2160 2.9087209 1.802355 -0.13447377 8.0660582 3.48274834 0.06201167
## Clade2164 2.3978953 1.620575 -0.20978808 7.3855425 3.17674400 0.07469343
## Clade2178 1.2992830 1.752343 -1.87143674 6.3832278 0.62341548 0.42978090
## Clade2251 2.3978953 1.726794 -0.51915821 7.4746607 2.51745400 0.11259224
## Clade2279 1.8101086 1.674527 -1.00799075 6.8377336 1.47868405 0.22398053
## Clade2286 1.8870696 1.802355 -1.36705766 7.0201279 1.27351077 0.25910909
## Clade2304 2.3978953 1.726794 -0.51915821 7.4746607 2.51745400 0.11259224
## Clade2313 0.6632942 1.722246 -2.45324441 5.7198019 0.16043161 0.68875946
## method
## (Intercept) 2
## Clade22 2
## Clade212 2
## Clade224 2
## Clade244 2
## Clade262 2
## Clade272 2
## Clade280 2
## Clade285 2
## Clade292 2
## Clade293 2
## Clade2132 2
## Clade2136 2
## Clade2159 2
## Clade2160 2
## Clade2164 2
## Clade2178 2
## Clade2251 2
## Clade2279 2
## Clade2286 2
## Clade2304 2
## Clade2313 2
##
## Method: 1-Wald, 2-Profile penalized log-likelihood, 3-None
##
## Likelihood ratio test=19.51261 on 21 df, p=0.5522927, n=111
## Wald test = 18.95332 on 21 df, p = 0.5881349

## Lower 95% Upper 95%
## (Intercept) -7.27518334 -0.2215505
## Clade22 -0.05189156 7.7856871
## Clade212 -1.00799075 6.8377336
## Clade224 -0.13447377 8.0660582
## Clade244 -1.46315378 6.3035109
## Clade262 -2.05167915 6.1718363
## Clade272 -1.36705766 7.0201279
## Clade280 -1.36705766 7.0201279
## Clade285 0.64208897 8.6139244
## Clade292 -1.65105598 6.6517090
## Clade293 0.23307460 7.7373133
## Clade2132 -0.13447377 8.0660582
## Clade2136 -4.87880089 5.7867551
## Clade2159 -0.51915821 7.4746607
## Clade2160 -0.13447377 8.0660582
## Clade2164 -0.20978808 7.3855425
## Clade2178 -1.87143674 6.3832278
## Clade2251 -0.51915821 7.4746607
## Clade2279 -1.00799075 6.8377336
## Clade2286 -1.36705766 7.0201279
## Clade2304 -0.51915821 7.4746607
## Clade2313 -2.45324441 5.7198019

## Setting levels: control = 0, case = 1

## Setting direction: controls < cases


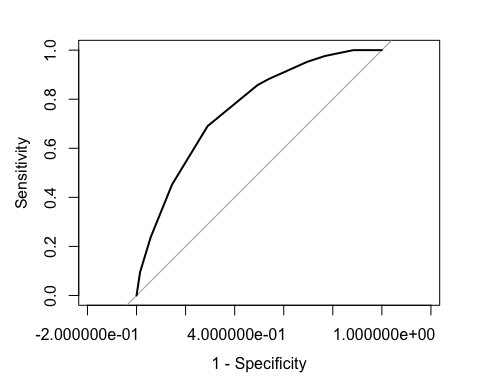


## Area under the curve: 0.7621

## OddsRatio OR_lower95 OR_upper95
## (Intercept) 0.09090909 0.0006925131 0.8012755
## Clade22 15.40000000 0.9494318172 2405.9186485
## Clade212 6.11111111 0.3649515221 932.3736402
## Clade224 18.33333333 0.8741758057 3184.5241880
## Clade244 3.66666667 0.2315050056 546.4872124
## Clade262 3.00000000 0.1285189198 479.0649940
## Clade272 6.60000000 0.2548557297 1118.9297011
## Clade280 6.60000000 0.2548557297 1118.9297011
## Clade285 33.00000000 1.9004467074 5507.8214621
## Clade292 4.71428571 0.1918472141 774.1061734
## Clade293 15.88888889 1.2624756560 2292.3054775
## Clade2132 18.33333333 0.8741758057 3184.5241880
## Clade2136 1.57142857 0.0076061292 325.9536177
## Clade2159 11.00000000 0.5950212222 1762.8034215
## Clade2160 18.33333333 0.8741758057 3184.5241880
## Clade2164 11.00000000 0.8107560443 1612.5023064
## Clade2178 3.66666667 0.1539023850 591.8349680
## Clade2251 11.00000000 0.5950212222 1762.8034215
## Clade2279 6.11111111 0.3649515221 932.3736402
## Clade2286 6.60000000 0.2548557297 1118.9297011
## Clade2304 11.00000000 0.5950212222 1762.8034215
## Clade2313 1.94117647 0.0860140682 304.8445348

## PercentChange PC_lower95 PC_upper95
## (Intercept) -90.90909 -99.930749 -19.87245
## Clade22 1440.00000 -5.056818 240491.86485
## Clade212 511.11111 -63.504848 93137.36402
## Clade224 1733.33333 -12.582419 318352.41880
## Clade244 266.66667 -76.849499 54548.72124
## Clade262 200.00000 -87.148108 47806.49940
## Clade272 560.00000 -74.514427 111792.97011
## Clade280 560.00000 -74.514427 111792.97011
## Clade285 3200.00000 90.044671 550682.14621
## Clade292 371.42857 -80.815279 77310.61734
## Clade293 1488.88889 26.247566 229130.54775
## Clade2132 1733.33333 -12.582419 318352.41880
## Clade2136 57.14286 -99.239387 32495.36177
## Clade2159 1000.00000 -40.497878 176180.34215
## Clade2160 1733.33333 -12.582419 318352.41880
## Clade2164 1000.00000 -18.924396 161150.23064
## Clade2178 266.66667 -84.609761 59083.49680
## Clade2251 1000.00000 -40.497878 176180.34215
## Clade2279 511.11111 -63.504848 93137.36402
## Clade2286 560.00000 -74.514427 111792.97011
## Clade2304 1000.00000 -40.497878 176180.34215
## Clade2313 94.11765 -91.398593 30384.45348

##
## Attaching package: 'MASS'

## The following object is masked from 'package:dplyr':
##
## select

##
## Re-fitting to get Hessian

## Call:
## polr(formula = RiskCategory ~ race, data = risk.2, method = "logistic")
##
## Coefficients:
## Value Std. Error t value
## raceAsian 0.5057 0.4312 1.1727
## raceBlack 1.2505 1.0360 1.2070
## raceHisp 0.2151 0.6663 0.3229
## raceNAAI 0.5057 0.5147 0.9824
## raceNHPI 1.1846 0.4149 2.8551
##
## Intercepts:
## Value Std. Error t value
## 0|1 -0.5197 0.2320 -2.2401
## 1|2 1.5310 0.2644 5.7901
##
## Residual Deviance: 325.6938
## AIC: 339.6938
## (251 observations deleted due to missingness)

## Waiting for profiling to be done...
##
## Re-fitting to get Hessian

## 2.5 % 97.5 %
## raceAsian -0.3384391 1.357389
## raceBlack -0.7974687 3.445917
## raceHisp -1.1091898 1.532198
## raceNAAI -0.5047168 1.523377
## raceNHPI 0.3795561 2.010805

## Waiting for profiling to be done...

##
## Re-fitting to get Hessian

## Waiting for profiling to be done...

##
## Re-fitting to get Hessian

## OddsRatio OR_lower95 OR_upper95
## raceAsian 1.658106 0.7128822 3.886034
## raceBlack 3.492189 0.4504678 31.372049
## raceHisp 1.240007 0.3298261 4.628339
## raceNAAI 1.658104 0.6036765 4.587692
## raceNHPI 3.269300 1.4616357 7.469330

## Waiting for profiling to be done...
##
## Re-fitting to get Hessian

## Waiting for profiling to be done...

##
## Re-fitting to get Hessian

## PercentChange PC_lower95 PC_upper95
## raceAsian 65.81061 -28.71178 288.6034
## raceBlack 249.21890 -54.95322 3037.2049
## raceHisp 24.00068 -67.01739 362.8339
## raceNAAI 65.81038 -39.63235 358.7692
## raceNHPI 226.92997 46.16357 646.9330
